# Mass spectrometric analysis of *Odonthobuthus Doriae* scorpion venom and its non-neutralized fractions after interaction with commercial antivenom

**DOI:** 10.1038/s41598-024-59150-z

**Published:** 2024-05-06

**Authors:** Adel Abdollahnia, Kiumars Bahmani, Atousa Aliahmadi, Mohammad Ali As’habi, Alireza Ghassempour

**Affiliations:** 1 Medicinal Plants and Drugs Research Institute, Shahid Beheshti University, G.C. Evin, Tehran, Iran; 2 Department of Pharmacology and Toxicology, School of Pharmacy, Shahid Beheshti University of Medical Sciences, Tehran, Iran

**Keywords:** Scorpion Venom, Antivenom, Neutralization, A0A5P8U2Q6_MESEU, A0A0U4FP89_ODODO, Mass Spectrometry, Pharmacology, Toxicology

## Abstract

It is believed that antivenoms play a crucial role in neutralizing venoms. However, uncontrolled clinical effects appear in patients stung by scorpions after the injection of antivenom. In this research, non-neutralized components of the venom of the Iranian scorpion *Odonthobuthus doriae* were analyzed after interacting with the commercial antivenom available in the market. The venom and antivenom interaction was performed, then centrifuged, and the supernatant was analyzed by high-performance liquid chromatography (HPLC). Two peaks of *Odonthobuthus doriae* venom were observed in the chromatogram of the supernatant. Two components were isolated by HPLC and analyzed by matrix-assisted laser desorption/ionization time-of-flight mass spectrometry (MALDI-TOF MS) instruments. Peptide sequencing was done by Liquid Chromatography Quadrupole Time-of-Flight Tandem Mass Spectrometry (LC-Q-TOF MS/MS). Results indicate that the components of scorpion venom mainly have a molecular weight below 10 kDa, consisting of toxic peptides that disrupt the function of sodium and potassium channels. The MALDI-TOF MS results show that two toxic peptides with molecular masses of 6941 Da and 6396 Da were not neutralized by the antivenom. According to the MS/MS sequencing data, the components have been related to peptides A0A5P8U2Q6_MESEU and A0A0U4FP89_ODODO, which belong to the sodium and potassium channels toxins family, respectively.

## Introduction

According to global health statistics, scorpion stings are a dangerous and neglected public health problem in countries with arid and semi-arid climates, especially in the Middle East^1^. Each year, scorpions cause 1.2 million stings, resulting in 3250 deaths^2^. About 50,000 cases of scorpion stings are registered in Iran annually. Epidemiological studies have shown that scorpion stings are one of the most common types of stings in Iran, most of which occur in the southern provinces^3^. The severity of scorpion envenomation depends more on the quantity of venom injected during the sting than on the number of stings, as sometimes a scorpion may sting without injecting venom^4^. However, the mortality rate of scorpion stings in Iran is reported to be 0.04–0.05% in the literature^5,6^.

Children with developing immune systems and elderly individuals who may have immunodeficiency are particularly vulnerable to the effects of a scorpion sting ^7^. After being stung by a scorpion, a person may experience a range of clinical manifestations and symptoms, which can vary in severity. These symptoms depend on factors such as the size and type of scorpion, the location and number of stings, as well as the age and overall health of the individual. General symptoms include severe local pain, numbness, sweating, tremors, and restlessness. In addition, individuals may experience abnormally fast breathing, an increased heart rate, and nausea. These symptoms are mostly caused by neurotoxins that affect ion channel modulators^8^.

Scorpions in Iran can be classified into three families in terms of molecular and morphological approaches, which include *Scorpionidae, Buthidae, and Hemiscorpiidae*^9^. The Iranian yellow scorpion, also known as *Odontobuthus doriae*, belongs to the *Buthidae* family and is distributed in most of Iran's provinces^10^. According to the median lethal dose (LD_50_), scorpions from the *Buthidae* family have the most clinical effects and are more toxic than other families^11^. Scorpion antivenom is the standard treatment for scorpion envenomation, which can reverse signs and symptoms caused by scorpion venom^12^. On the other hand, in the treatment of scorpion stings envenomation, efficacy and quality of the antivenom play crucial roles, because an effective antivenom significantly decreases the level of circulating unbound venom within a few hours^13^.

There are numerous techniques available to study venom, antivenom compositions, and antivenom efficacy. One prominent technique is venomics, which focuses on comprehensively analyzing and identifying the various components present in venom and investigating their respective functions^14^. Antivenomics is the study of the interaction between venom and antivenom, including the identification of the components of antivenom that bind to venom components^14,15^. Various techniques are utilized for venomics and antivenomics studies including reversed-phase high-performance liquid chromatography (RP-HPLC), sodium dodecyl sulfate polyacrylamide gel electrophoresis (SDS-PAGE) separation, matrix-assisted laser desorption/ionization time-of-flight mass spectrometry (MALDI-TOF MS), and LC–MS/MS for separation and identification of venom toxins^16^.

Given the presence of *Odontobuthus doriae* scorpion in our area and high rates of envenomation by this scorpion it was selected for this study. It has been shown that low molecular weight toxins are poorly immune-recognized by commercial scorpion antivenom^2^. The aim of this study is to investigate and identify the components of scorpion venom and analyze their interaction with the commercially available antivenom in our local market. The goal is to identify the toxins that are not neutralized by antivenom antibodies and determine the efficacy of the commercial antivenom.

## Methods and materials

### Solvents and reagents

The α-4-hydroxycinnamic matrix, proteomics grade trypsin, trifluoroacetic acid (TFA) and acetonitrile (ACN) with HPLC purity were purchased from Sigma Aldrich (Sigma-Aldrich Corporation. a subsidiary of Merck KGaA, Darmstadt, Germany). Ammonium bicarbonate, dithiothreitol (DTT) and iodoacetamide were also obtained from Sigma Aldrich. Deionized water was prepared using a Milli-Q system and was used for the preparation of all buffers.

### Preparation of venom sample

#### Scorpions

The *Odontobuthus doria* scorpion, belonging to the Buthidae family, was obtained from a scorpion breeding farm in Larestan, Fars province, Iran*.*

#### Venom milking

The crude venom was obtained through electrical stimulation of the scorpion's telson using an electropulse stimulator with a voltage between 7 and 10 V. A total of 200 *Odontobuthus doriae* scorpions, including both male and female specimens were used. The extracted venom which had a milky white color, was transferred to sterile vials and stored in a liquid nitrogen tank until it was brought to the laboratory^17^.

#### Freez drying

First, the collected venom was lyophilized for 48 h using Alpha 1 (Martin Christ GmbH, Osterode am Harz, Germany). It was then stored in a -20 °C freezer for further analysis.

### Determination of venom protein concentration

To determine the concentration of the scorpion venom protein being studied, the Bradford assay was utilized. A six-point calibration curve ranging from 0.2 to 2 μg/μL was created using a BSA standard solution. Venom samples were diluted for analysis, and then 1.5 mL of Bradford's reagent was added to all samples. After 20 min, absorbance was measured at 595 nm. The standard sample was prepared in duplicate, and the mean results were used for standard curve preparation. The samples were then analyzed in triplicate.

The protein concentration of the venom fractions was also measured using this method before MALDI-TOF MS analysis or digestion.

### Determination of the median lethal dose (LD_50_) of venom samples and median effective dose (ED_50_) of antivenom

The lethal potency (LD_50_) of scorpion venom (in micrograms of lyophilized venom per gram for mice) was determined according to the World Health Organization (WHO) guidelines^18,19^. Briefly, six male BALB/c mice weighing about 18–20 g per group were selected for each dose of venom. The venom was diluted in injectable 0.9% NaCl solution to obtain various concentrations (25 μg/mL, 50 μg/mL, 100 μg/mL, and 200 μg/mL) and injected intravenously into the tail vein with a final volume of 200 μL. The control group was injected with 200 μL of 0.9% NaCl as the carrier of venom. Mortality was recorded 24 h after injection. The average lethal dose was calculated by Prism 9 GraphPad software package using non-linear curve fitting (variable slope) and a four-parameter logistic equation, with limits imposed on minimum (0% mortality) and maximum (100% mortality) values.

The ED_50_ of antivenom for neutralization of venom lethality was determined by mixing a challenge dose of venom containing 3 LD_50_ of venom with various amounts of antivenom (2.5, 5, 10, 15, and 20 μL) then incubated for 30 min at 37 °C. For the control group one animal was selected and the venom challenge dose was mixed with 0.9% NaCl and incubated with the other samples. The samples were then centrifuged at 10,000 × g for 12 min, and 200 μL of supernatant was injected intravenously into the tail vein. Mortality was recorded 24 h after injection. After the test, the animals that were injected with venom were anesthetized by placing them in the isoflurane chamber, then euthanasia was performed by cervical dislocation.

### Antivenom production and preparation

The commercial antivenom against the venoms of medically important scorpions including *Androctonus crassicauda, Mesobuthus eupeus, Odontobuthus doriae, Hottentotta saulcyi, Hottentotta schach* and *Hemiscorpius lepturus* was used in this study (PadraSerum company, Alborz, Iran. https://www.padraserum.com accessed 09/13/2023).

### Venom and antivenom interaction

In this study, according to previous research in the field of interaction between venom and antivenom^20^, we utilized an antivenom to venom ratio of 10:1 (based on protein concentration)^21^. The mixture was then vortexed for 1 min in the ambient environment, followed by incubation in a 37 °C environment for 30 min. Subsequently, the mixture was centrifuged for 10 min at 12,000 × g. The resulting supernatant was transferred to a microtube for further analysis.

### Chromatographic separation

#### RP-HPLC

One mg of lyophilized crude venom was dissolved in 1 mL deionized water to extract the components (proteins and peptides) of the mixed venom, remove its mucus and lipids, and then centrifuged at 10,000 × g, 4 °C for 10 min. Subsequently, the supernatant containing soluble venom proteins was collected and filtered (Millex-HV, 0.45 µm, Millipore, Merck KGaA, Darmstadt, Germany) for HPLC analysis. Scorpion venom peptides were finally separated by an Agilent LC 1100 HPLC apparatus (Agilent Technologies, Santa Clara, California, USA) with a gradient system equipped with a DAD detector after sample preparation. The injection of 20 µL of the sample with a concentration of 1000 µg/mL was performed manually with a 100 µL loop. In this section, the C18 column 4.6 × 250 mm, 5 µm, 100 Å, (Knauer Berlin, Germany) was used for separation. The gradient program including solvent A (H_2_O and 0.1% TFA) and solvent B (ACN 0.1% TFA) was applied with a flow rate of 0.5 mL/min and the chromatograms were recorded at the wavelength of 215 nm using a DAD detector. The column wash program used was a linear gradient from 5 to 30% ACN with 0.1% TFA in 60 min, followed by a gradient from 30 to 70% ACN with 0.1% TFA. The method was optimized in 140 min. The same method and conditions were employed for antivenom analysis.

#### The interaction of venom and antivenom and RP-HPLC analysis

Interaction of venom with antivenom was conducted with a ratio of 1/10 as protein concentration then incubated at 37 °C for 30 min. The mixture was then centrifuged at 10,000 × g for 12 min and the supernatant was collected for HPLC, MALDI-TOF MS and LC–MS/MS analysis. HPLC of the supernatant was performed using the same method for crude venom and antivenom, and peaks were collected and lyophilized for MALDI-TOF MS analysis.

### Mass spectrometry and identification of venom components

#### MALDI-TOF MS

For the analysis of venom, antivenom, and their interaction, peaks were collected after HPLC analysis. One microliter of each was mixed with 2 µl of α-cyano-4-hydroxycinnamic acid (1:2 v/v) in H_2_O/ACN containing 0.1% TFA and vortexed. To homogenize, 1μL of this mixture was placed on a special MALDI-TOF MS plate and allowed to dry at room temperature. The device used for analysis was the Applied Biosystems 4800 MALDI TOF with a Nd: YAG 200-HZ laser (SCIEX, Redwood City, California, USA) in linear positive mode. The spectrum was obtained after 1600 laser shots, and Data Explorer software version 4.0 from Applied Biosystems (Waltham, Massachusetts, United States) was used for data interpretation and processing.

#### Cut off filter

Microcon-10 kDa centrifugal filter was obtained from Merck (Merck KGaA, Darmstadt, Germany) and was utilized to concentrate venom proteins that are 10 kDa and lower for liquid chromatography- tandem mass spectrometry.

#### In solution digestion

The protein concentration of fractions was measured as described in the section "Determination of Venom Protein Concentration". then in solution digestion performed for the collected fractions as previously described^22^ with modification in enzyme to protein ratio (25:1). The trypsin-digested peptides were desalted using ZipTip® Pipette Tips C18 resin (Merck Millipore, Burlington, Massachusetts, United States).

#### Electrospray ionization tandem mass spectrometry

A Synapt G1 HDMS LC-ESI-qTOF MS/MS system from Waters (Milford, Massachusetts, USA) coupled to an Agilent 1100 HPLC system was utilized to analyze the venom fraction lower than 10 kDa and the fractions obtained from the RP-HPLC of antivenom-venom interaction. The venom fraction lower than 10 kDa, at a concentration of 1000 µg/mL, and digested fractions were prepared for analysis and injected into a C18 reverse-phase column (4.6 × 250 mm, 5 µm, 100 Å, Kherad Azma Tehran, Iran) with the same settings as described in the RP-HPLC section, except that trifluoroacetic acid (TFA) was replaced with formic acid (FA). The eluate was then sent to the ESI-qTOF MS/MS system under the following conditions: Ionization mode: ESI positive mode, Capillary voltage: 2.1 kV, Cone voltage: 5 kV, Detector voltage: 2 kV, Acquisition mode: V-mode (> 10,000), Acquisition rate: Low, and elevated energy functions at 0.5 s, Collision energy: 5 eV (low energy function); 20–45 eV linear collision energy ramp (elevated energy function), Source temperature: 120 °C, desolvation temperature: 500 °C, desolvation gas flow: 600 L/hr, and cone gas flow: 50 L/hr. MS calibration was performed using sodium iodide. The full scan mass spectrometry had a duration of 140 min with a cycle time of 1.012 s (total of 8300 cycles) and data acquisition was done using MassLynx 4.1 software (Waters, Milford, Massachusetts, USA) with a scan range of 50–4000 m/z.

The MS/MS fragmentation was done using CID and data acquisition was done using MassLynx 4.1 software (Waters, Milford, Massachusetts, USA) with scan range of 20–3000 m/. TOF resolution in positive ion mode was 10,000 FWHM in sensitivity mode, 20,000 FWHM in resolution mode, and 40,000 FWHM in high resolution mode.

The MS data and deconvolution were also done using MassLynx 4.1 software (Waters, Milford, Massachusetts, USA).

### Data analysis

In this study, the raw data from LC–MS/MS and MALDI-TOF MS were analyzed using databases related to the Buthidae family (Taxid: 6555) of NCBI and UniProt, which are relevant to the studied scorpion. To achieve this, the Pyteomics package in the Python programming language was employed^23^.

Our analysis is structured as follows:

The raw data was compiled with consideration of the intensity filtration of qualified ions as input. Then, bottom-up proteomics analysis was applied to the input data. The data processed in the aforementioned analysis was initially presented as a sequence of peptide fragments with a mass error of 1.2 ppm and a false discovery rate (FDR) of < 1%. Furthermore, their maximum coverage was interpolated based on NCBI (https://www.ncbi.nlm.nih.gov// accessed on 10/23/2023) and UniProt (https://www.uniprot.org accessed on 10/23/2023) databases of the Buthidae family (Taxid: 6555).

#### Ethics

All procedures related to animals were conducted in accordance with ethical principles in animal research, with permission from the Ethics Committee of Shahid Beheshti University and approval code IR.SBU.REC.1402.040. In addition, all study procedures are reported in accordance with ARRIVE guidelines (https://arriveguidelines.org).

## Results and discussion

### Determination of protein concentration

The Bradford test results demonstrated that the protein content of crude venom is approximately 870 µg/mg, while the protein content of antivenom is about 50 mg/ml. The concentration of fractions was found to be 19 µg/mL and 15 µg/mL respectively.

### Investigating the median lethal dose of scorpion venom and median effective dose of antivenom

The results of the median lethal dose test revealed an LD_50_ of 1.075 µg/g for mice. The venom neutralization efficacy test (ED_50_) was performed on the antivenom using a fixed amount of venom mixed with different volumes of antivenom. The obtained LD_50_ is lower than the LD50 of some other scorpions like *Hemiscorpius lepturus* which was reported to be 177.01 µg/mouse in one study^24^ and 107±26.75 µg/mouse in another study^25^. This difference in venom lethality could be due to the higher toxicity of *Odontobuthus doriae* venom compared to *Hemiscorpius lepturus.* These results are consistent with the literature on the venom toxicity of medically important scorpions in Iran^26^.

**Figure 1:**
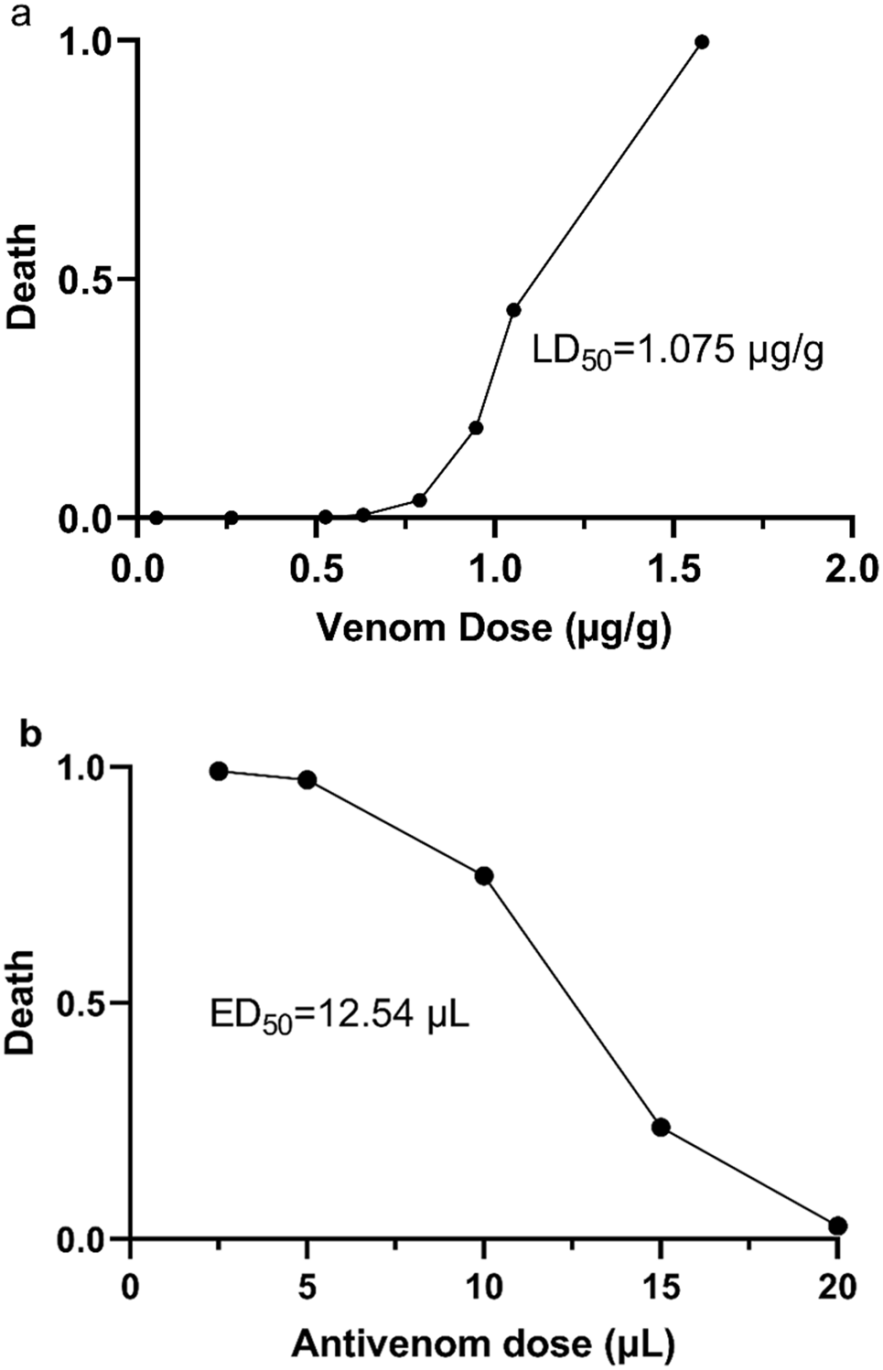
(**a**) LD_50_ of crude venom of *Odontobuthus doriae*. (**b**) ED_50_ of antivenom for neutralization of venom lethality.

ED_50_ is the initial stage of the WHO protocol used to determine the effectiveness of an antivenom in neutralizing the lethal effects of scorpion venom^27,28^. The results of the neutralization of lethality test showed that the ED_50_ is 12.54 µL of antivenom for a challenge dose of 3LD_50_ of venom, as recommended by WHO guidelines^18^. The graphs related to the venom LD_50_ and the antivenom ED_50_ tests are shown in Fig. 1.

### Chromatographic profile of venom, antivenom and their interactions

**Figure 2:**
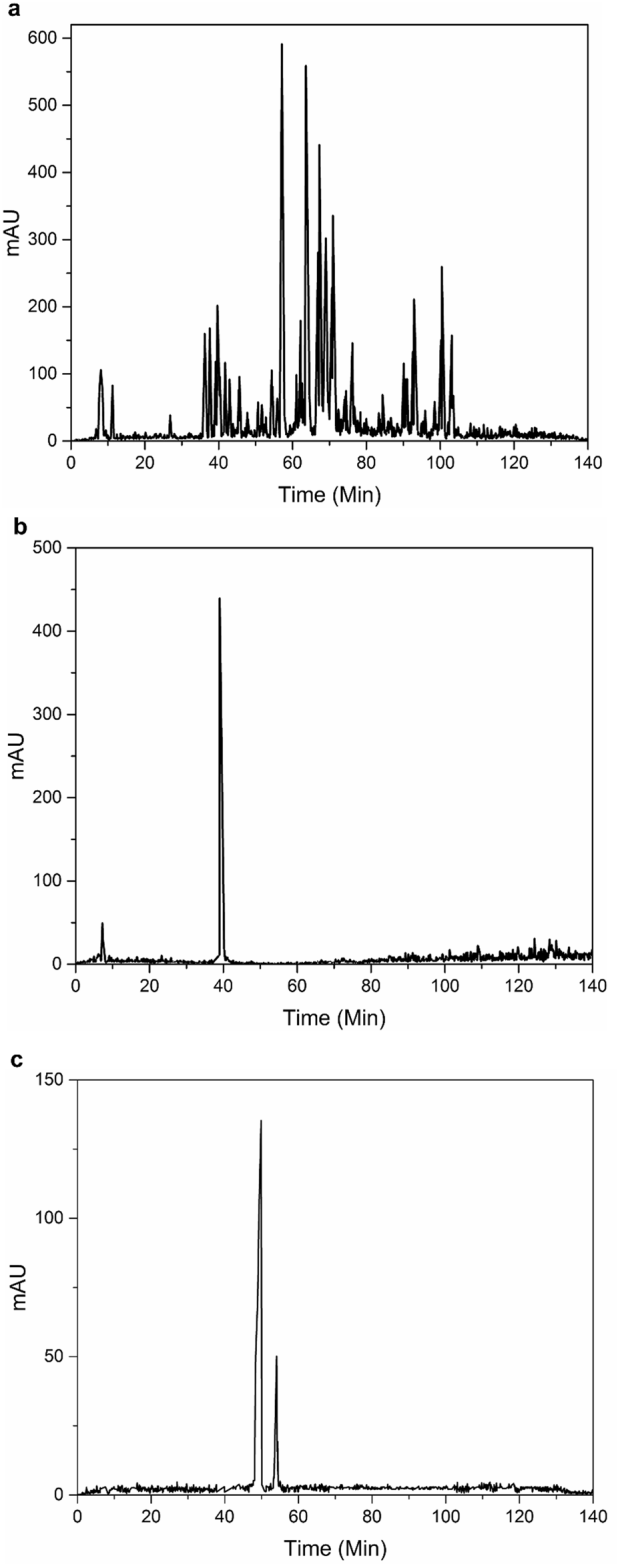
High-performance liquid chromatography chromatograms obtained using C18 column under gradient wash of ACN/H_2_O for 140 min for crude venom (**a**), antivenom (**b**), and the supernatant of venom and antivenom interaction (**c**).

The chromatographic profile obtained, as shown in Fig. 2A, reveals that the crude venom has 38 significant peaks with the highest intensity obtained for the peak at 58 min retention time and the peak at 84 min retention time having the lowest intensity. The complex composition of *Odonthobuthus doriae* venom in this study is consistent with existing literature^28,29^. Previous studies on other scorpion species have also described the same complexity of chromatographic profiles, including *Androctonus mauretanicus mauretanicus*, *Androctonus crassicauda*, *Androctonus bicolor*, *Leiurus quinquestriatus*, and *Tityus serrulatus*^28,30,31^. The RP-HPLC chromatogram of the antivenom showed two peaks (Fig. 2b). The first peak is related to the preservative, while the second peak, with higher intensity, is related to the F(ab')2 antibody fragments. Other studies on purified F(ab')2 antibody fragments have also reported similar results^32^. Figure 2c shows that the RP-HPLC profile changed after interaction, with most of the peaks in the venom being removed. However, two peaks at the RT of 49 and 54 min remained. The changes in the HPLC profile of scorpion venom after interaction may be due to interactions with antivenom F(ab')2 antibody fragments. The suppression of most venom peaks may indicate the neutralization of venom components by the antivenom. The two remaining peaks may be due to the presence of venom components that were not completely neutralized by the antivenom^24^.

### Mass spectrometry results

#### Mass spectrometric profile of crude venom

The washing profile of venom peaks from the total ion chromatogram (TIC), the heatmap of MS1 created using SeeMS software (https://proteowizard.sourceforge.io/), and the 3D visualization of MS1 created using Mass++ software (http://groups.google.com/group/massplusplus/) are presented in Supplementary Figure S1. These visual representations provide an insight into the elution pattern of the venom components. Additionally, Table 1 displays the masses of the components of the fractions obtained during the LC-MS analysis.

**Table 1:** The masses of the components of the fractions obtained during the liquid chromatography-mass spectrometry analysis. The fractions were collected at 10 min intervals within the time period of 0–140 min. The mass profile of the scorpion *Odontobuthus doriae*, where the signal intensity of molecular masses greater than 1 × 10*4 was identified and reported.

RT (Min)	M, Mass (Da)
0–10	149.02, 198.19, 213.18, 3367.14, 2845.82, 1902.32, 262.97, 871.57, 458.93, 542.92, 430.93
10–20	268.11, 1482.95, 2085.65
20–30	1485.29, 2478.37, 261.13, 1782.11, 3180.30, 726.85, 494.26, 1016.18, 2517.77, 3656.55
30–40	1912.43, 802.87, 319.15, 305.16, 993.42, 289.13, 608.77, 363.18, 770.31, 349.20, 2421.06, 407.21, 1225.36, 988.44, 1482.69, 1521.13, 333.16
40–50	1061.79, 407.19, 796.59, 393.22, 1020.24, 1359.67, 1432.72, 999.21, 1332.28, 377.19, 763.45, 437.25, 1134.84, 495.25
50–60	481.27, 812.44, 539.29, 1380.36, 1035.74, 525.30, 451.25, 1453.70, 611.30, 1090.73, 569.33, 1294.31, 970.68, 495.29, 613.37, 553.29, 1289.00
60–70	539.31, 1165.49, 1744.63, 652.42, 1165.19, 2326.40, 597.32, 578.39, 578.39, 701.40, 518.34, 641.36, 1239.55, 1270.88, 1883.78, 622.43, 953.38, 562.37
70–80	671.39, 1223.19, 917.39, 1835.39, 710.47, 369.21, 577.32, 505.28, 1802.72
80–90	413.23, 754.53, 621.33, 549.33, 1442.32, 457.26, 1687.95
90–100	390.19,545.32, 1741.63, 841.49, 1727.89
100–110	435.22, 589.34, 633.36, 479.24, 667.42, 1720.62, 523.27, 1679.31
110–120	567.29, 2023.95, 611.33, 655.36, 699.38, 759.45
120–130	569.3, 613.34, 657.36, 696.45, 561.27, 649.335
130–140	693.38, 737.38, 553.25, 597.29, 641.30,431.27

#### MALDI-TOF MS profile of crude venom, and their interaction

**Figure 3:**
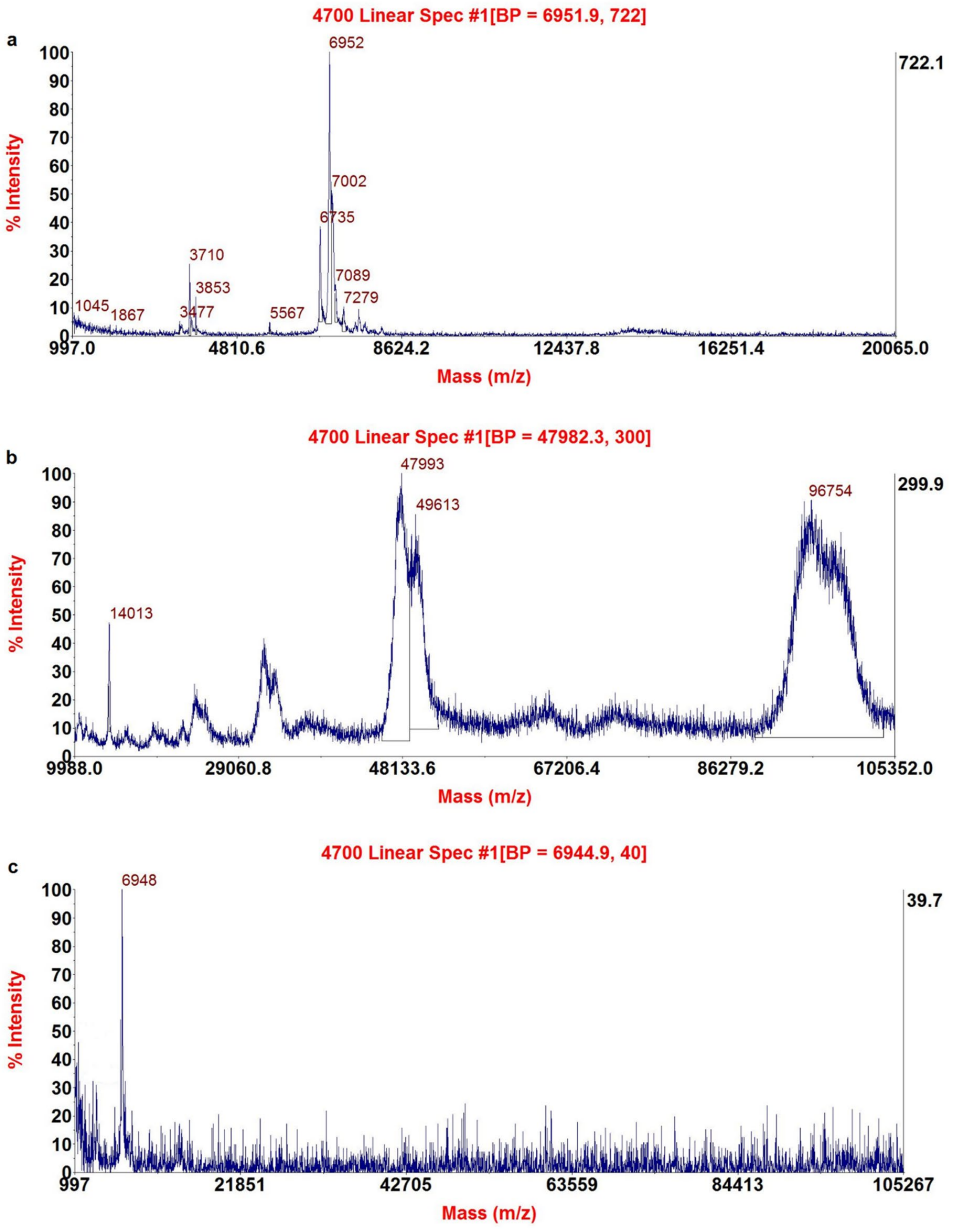
Mass profile using MALDI-TOF MS device: crude venom (**a**), antivenom (**b**), and the supernatant of their interaction in a ratio of 1:10 (**c**)**.**

The results obtained from the MALDI-TOF MS analysis showed 13 dominant molecular masses highlighted without the use of separation methods (Fig. 3a). Other studies have also shown the complex nature of scorpion venom in MALDI-TOF MS analysis^33,34^. Figure 3b shows the MALDI-TOF MS profile of antivenom, which has two prominent masses: the first one is about 100 kDa corresponding to F(ab')2 and the second one is about 50 kDa corresponding to Fab immunoglobulin fragments or heavy chain^35^.

Interestingly, a peptide with a mass of 6948 Da was present after the interaction of venom and antivenom (see Fig. 3c). However, since we observed two peaks in RP-HPLC analysis of the post-interaction supernatant (Fig. 2c), we collected the fractions for further analysis by LC-MS/MS as was done in our previous study^36^.

Since antivenoms are produced by animal immunization, there may be toxins in the venom with low immunogenicity to animals^37^. This could be a peptide to which the antibodies have a lower affinity to bind. Studies have shown lower immunogenicity for low molecular weight toxins in venoms^38,39^. Therefore, this result could be due to the low molecular mass of the remaining peptide after the interaction of venom with the antivenom.

The areas of molecular mass identification are also highlighted, allowing for more accurate analyses in the areas where there are the largest number of molecular masses, as shown in Fig. 3.

#### Identification of venom peptides

**Figure 4:**
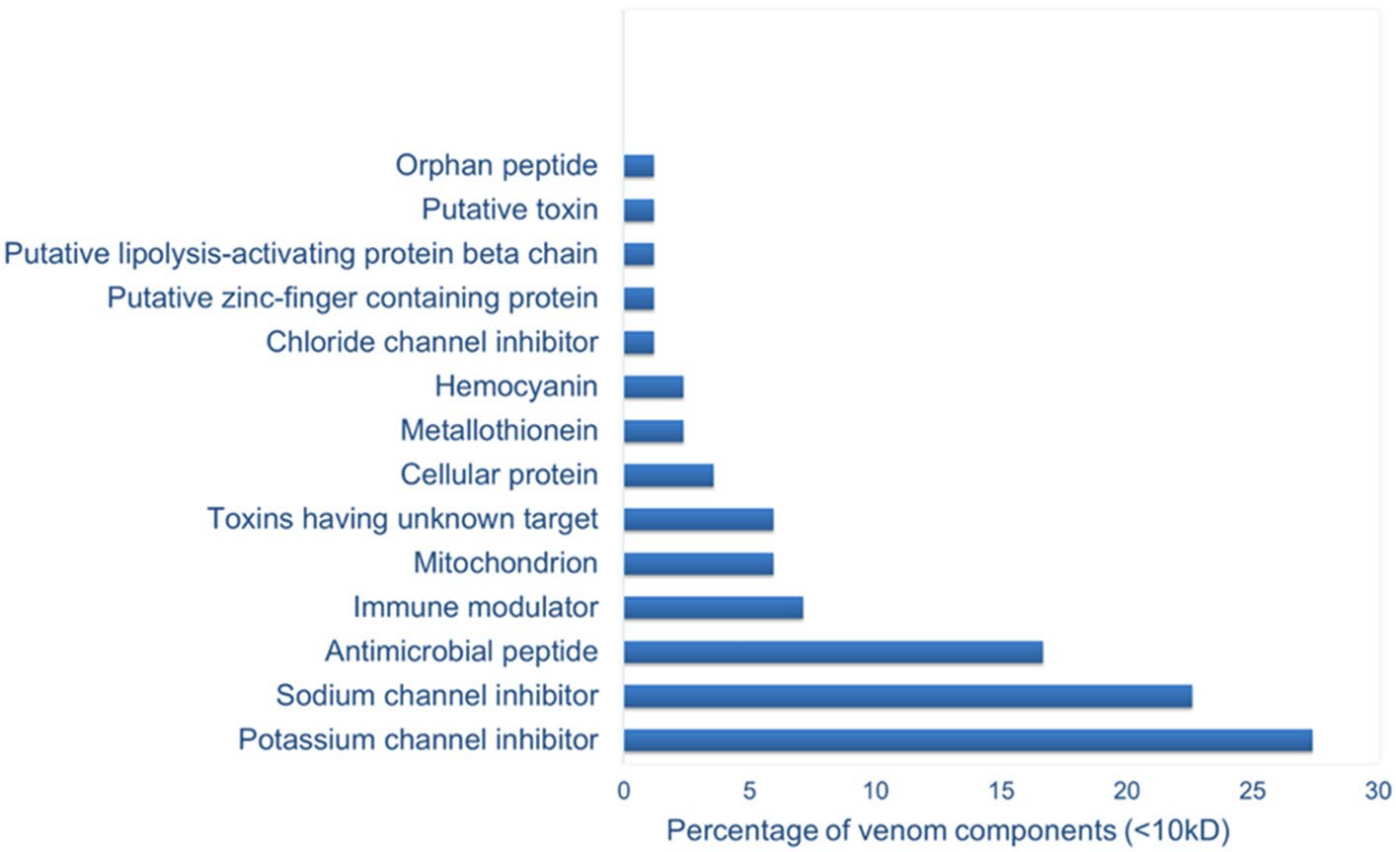
Composition of *Odontobotus doria* scorpion venom 10 kDa filtrate. The relative abundance of different venom protein families was calculated as the mean relative abundance.

Since the non-neutralized masses were under 10 kDa in molecular weight, a cut off filter was used to concentrate and separate the venom peptides with a molecular weight under 10 kDa. The results of LC-MS/MS showed that most of these peptides belong to two known families previously discovered in the venom of other scorpion species: Na^+^ and K^+^ channel toxins. According to the literature, scorpion venom contains a high proportion of peptide toxins that act by modulating voltage-gated Na^+^/K^+^ channel activity^40^. Here, we analyzed the data obtained from MS/MS using an algorithm designed with the Python programming language. Peptide identification was done with an approximation, due to high sequence similarities found in other venoms. The data were expressed as percentages and identified components were reported (see Fig. 4 and Table S1). However relative abundances and the percentages of the described peptides/proteins are purely based on total number counts. Studies on scorpion venoms have determined that it contains phospholipases A2, serine proteases, metalloproteases, lipolysis activating peptides, hyaluronidases, proteins, and several peptide toxins^9^. However, in our study some of these components were not observed because the reported composition is related to the analysis of the 10 kDa filtrate.

**Figure 5:**
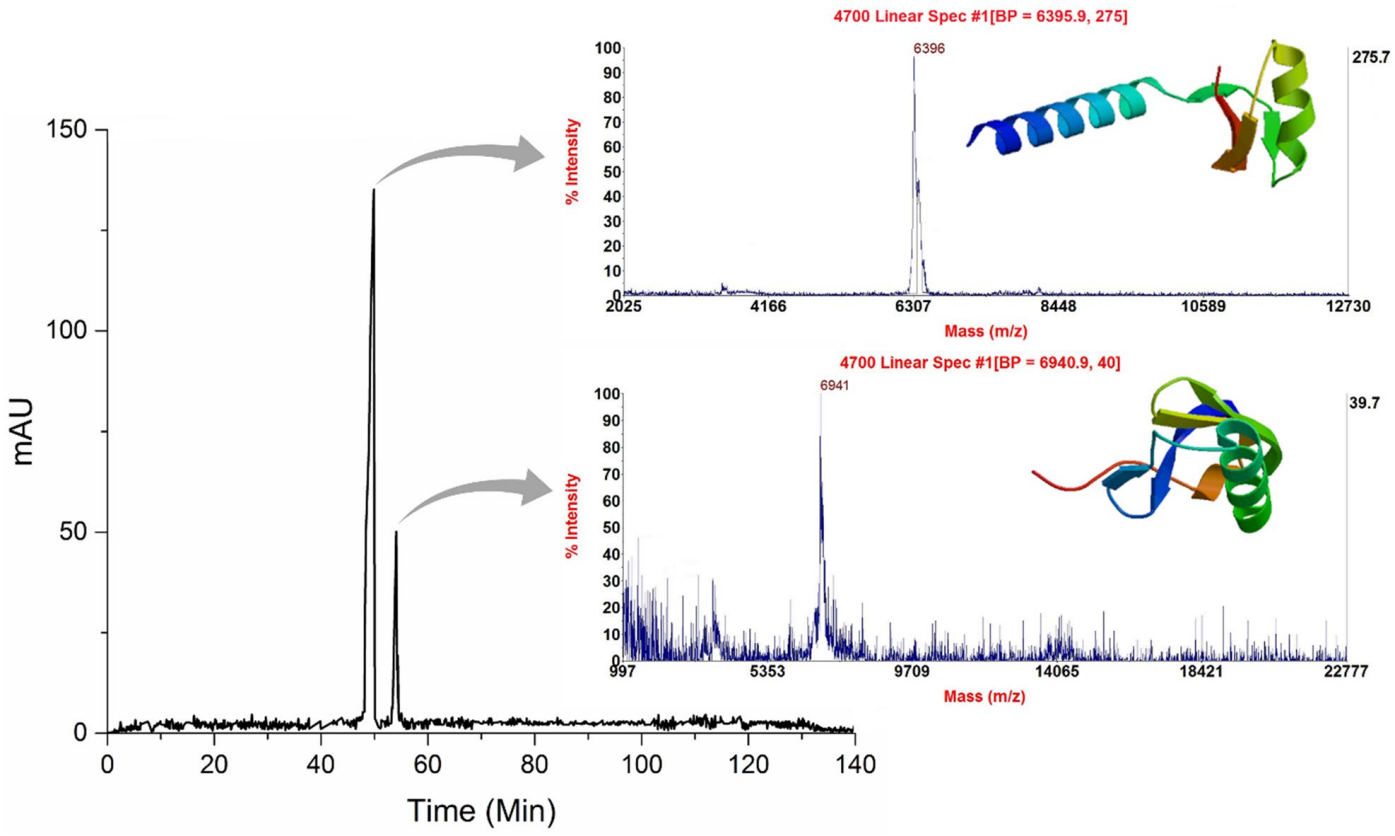
The Un-neutralized components were obtained from the interaction of venom and antivenom 1:10 and their different ratios with their mass spectrometry analysis. These components have a weight lower than 10 kDa.

The results of HPLC analysis of the supernatant obtained from the interaction of the venom and antivenom are presented in Fig. 5. It is evident that there are two peaks despite the MALDI-TOF MS results indicating a single mass. To further investigate this discrepancy, we collected fractions from the HPLC analysis of the supernatant and lyophilized them for MALDI-TOF MS analysis. This discrepancy may be attributed to the presence of multiple peptides in the analyte, as the presence of one analyte can suppress the ionization of another analyte in the same sample, especially in complex samples^41,42^.

The MALDI-TOF MS results of the collected fractions revealed two peptides with molecular masses of 6396 Da and 6941 Da, respectively (see Fig. 5). The peptide with a mass of 6941 Da was identified as a neurotoxin with sodium channel inhibitory activity, while the second peptide with a mass of 6396 Da was identified as a toxin with potassium channel inhibitory activity (see Table 2). These results are consistent with the literature regarding the molecular masses of the aforementioned toxins^43,44^.

**Table 2:** Analysis of the un-neutralized components of the scorpion *Odontobotus* doria venom using MALDI-TOF MS device for components with a molecular mass below 10 kDa.

Accession	Description	Organism	Coverage	Avg. Mass
Na +—channel toxins				
A0A5P8U2Q6_MESEU	Neurotoxin	Mesobuthus eupeus	59	6941
k +—channel toxins				
A0A0U4FP89_ODODO	Potassium channel toxin KTx5	Odontobuthus doriae	59	6396

## Conclusion

The analysis of scorpion venom revealed that not all toxins in the complex mixture were neutralized after interaction with antivenom. Among the remaining peptides, one was a sodium channel inhibitor toxin and the other was a potassium channel inhibitor toxin, both with low molecular weight. The study also found that interactions with antivenom reduced the abundance of these toxins. Future studies should consider investigating protein recombinant neutralizing antibodies and the phage display technique for these toxins.

### Supplementary Information

Supplementary Information.

## Data Availability

Data are available upon request from corresponding author.

## Abbreviations

ACN: Acetonitrile
ARRIVE: Animal research: reporting of in vivo experiments
BALB/c: Bagg albino
Da: Dalton
DAD: Diode array detector
DTT: Dithiothreitol
ED50: Effective dose for 50% of the population
kDa: Kilodalton
LD50: Median lethal dose
NCBI: National center for biotechnology information
RP-HPLC: Reversed-phase liquid chromatography
RT: Retention time
SDS-PAGE: Sodium dodecyl-sulfate polyacrylamide gel electrophoresis
MALDI-TOF MS: Matrix assisted laser desorption/ionization-time-of-flight mass spectrometry
LC–MS/MS: Liquid Chromatography with tandem mass spectrometry
QTOF- MS/MS: Quadrupole time-of-flight tandem mass spectrometry
TIC: Total Ion chromatogram
TFA: Trifluoroacetic acid
WHO: World Health Organization
