# Supplementary material for: Mass spectrometric analysis of Odonthobuthus Doriae scorpion venom and its non-neutralized fractions after interaction with commercial antivenom
Source: Sci Rep. 2024 May 6;14:10389. doi: 10.1038/s41598-024-59150-z (PMC11074270; doi:10.1038/s41598-024-59150-z)
Supplement: Supplementary file 1 — Supplementary Information. [file 41598_2024_59150_MOESM1_ESM.pdf]

## Supporting information

**Title:**

Mass Spectrometric Analysis of Odonthobuthus Doriae Scorpion Venom and Its Non-neutralized Fractions After Interaction with Commercial Antivenom

**Names of authors:**

Adel Abdollahnia[1], Kiumars Bahmani[2], Atousa Aliahmadi[1], Mohammad Ali As'habi[1], Alireza Ghassempour[1,*].

[1] Medicinal Plants and Drugs Research Institute, Shahid Beheshti University, G.C., Evin, Tehran, Iran.

[2] Department of Pharmacology and Toxicology, School of Pharmacy, Shahid Beheshti University of Medical Sciences, Tehran, Iran

* To whom correspondence should be addressed:
Alireza Ghassempour
Department of Phytochemistry, Medicinal Plants and Drugs Research Institute, Shahid Beheshti University, G.C.,
Evin, Tehran, Iran.
Tel. / fax: +982122431598
E-mail: a-ghassempour@sbu.ac.ir

***Corresponding Author:**

* To whom correspondence should be addressed:
Alireza Ghassempour
Department of Phytochemistry, Medicinal Plants and Drugs Research Institute, Shahid Beheshti University, G.C.,
Evin, Tehran, Iran.
Tel. / fax: +982122431598
E-mail: a-ghassempour@sbu.ac.ir

| Sl. No. | Name | Brief caption |
|---|---|---|
| ١ | Supplementary Fig S1 | Mass profiling of *Odontobuthus doriae* venom. Total ion chromatogram (TIC) of the soluble venom on the C18 column under a ACN/H2O gradient elution for 140 min (MassLynx 4.1) and The washing profile of venom peaks from the total ion chromatogram (TIC), heatmap of MS1 (using |

| | | |
|---|---|---|
| | | SeeMS software) and 3D visualization of MS1 (using Mass++ software)) provides visual representation of the elution pattern of the venom components. |
| 2 | Supplementary Fig S1 | MS/MS fragmentation and peptide sequencing of collected from the RP-HPLC of supernatant of antivenom-venom interaction |
| 3 | Supplementary Table S1 | The masses of the fractions obtained during the liquid chromatography-mass spectrometry analysis. The fractions were collected at 10-minute intervals within the time period of 0 to 140 minutes. The mass profile of the scorpion *Odontobuthus doriae*, where the signal intensity of molecular masses greater than 1×10*4 was identified and reported. |

**Supplementary Fig S1**. Mass profiling of *Odontobuthus doriae* venom. Total ion chromatogram (TIC) of the soluble venom on the C18 column under a ACN/H2O gradient elution for 140 min (MassLynx 4.1) and The washing profile of venom peaks from the (**a**) total ion chromatogram (TIC), (**b**) heatmap of MS1 (using SeeMS software) and (**c**) 3D visualization of MS1 (using Mass++ software) provides visual representation of the elution pattern of the venom components.

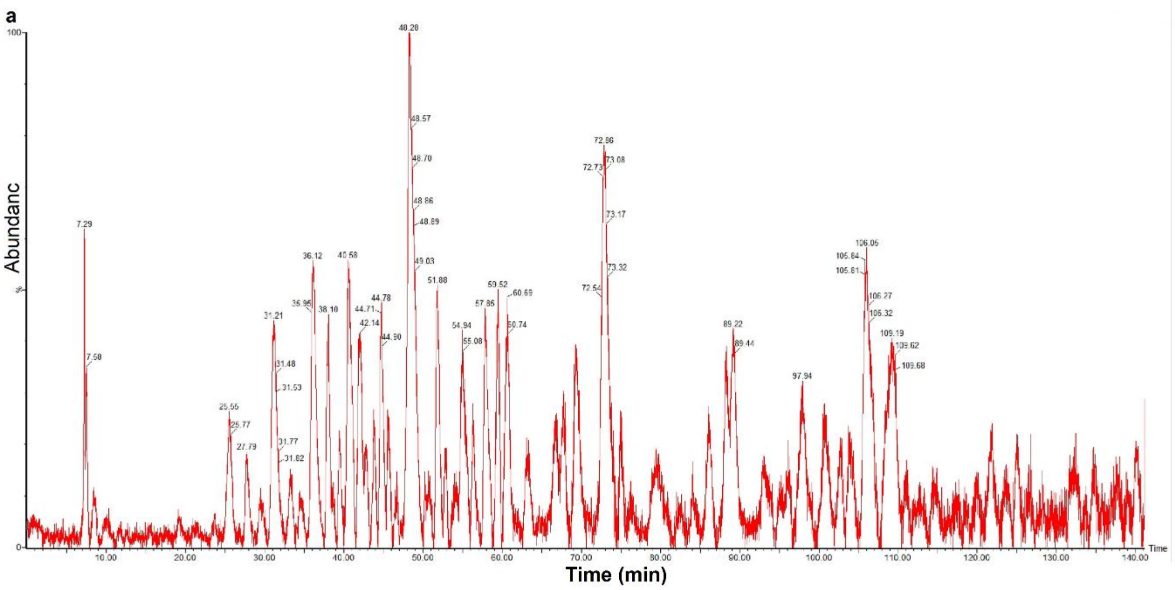

b

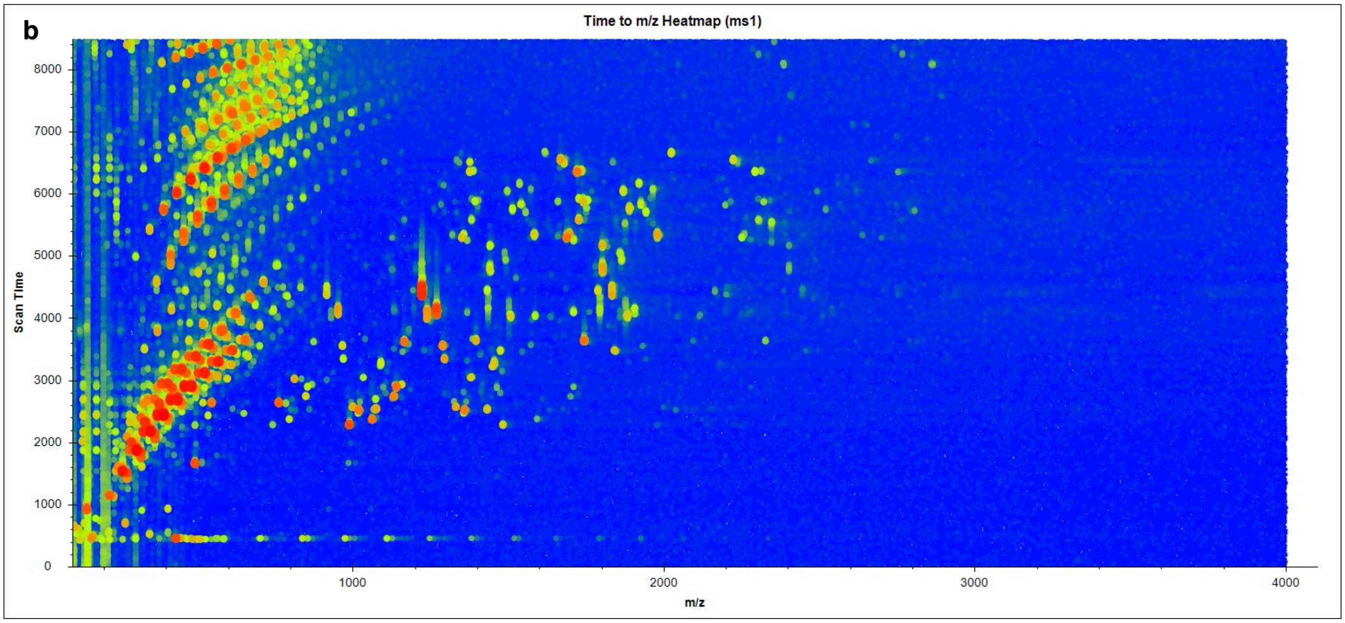
Time to m/z Heatmap (ms1)
Scan Time
8000
7000
6000
5000
4000
3000
2000
1000
0
1000
2000
3000
4000
m/z

c

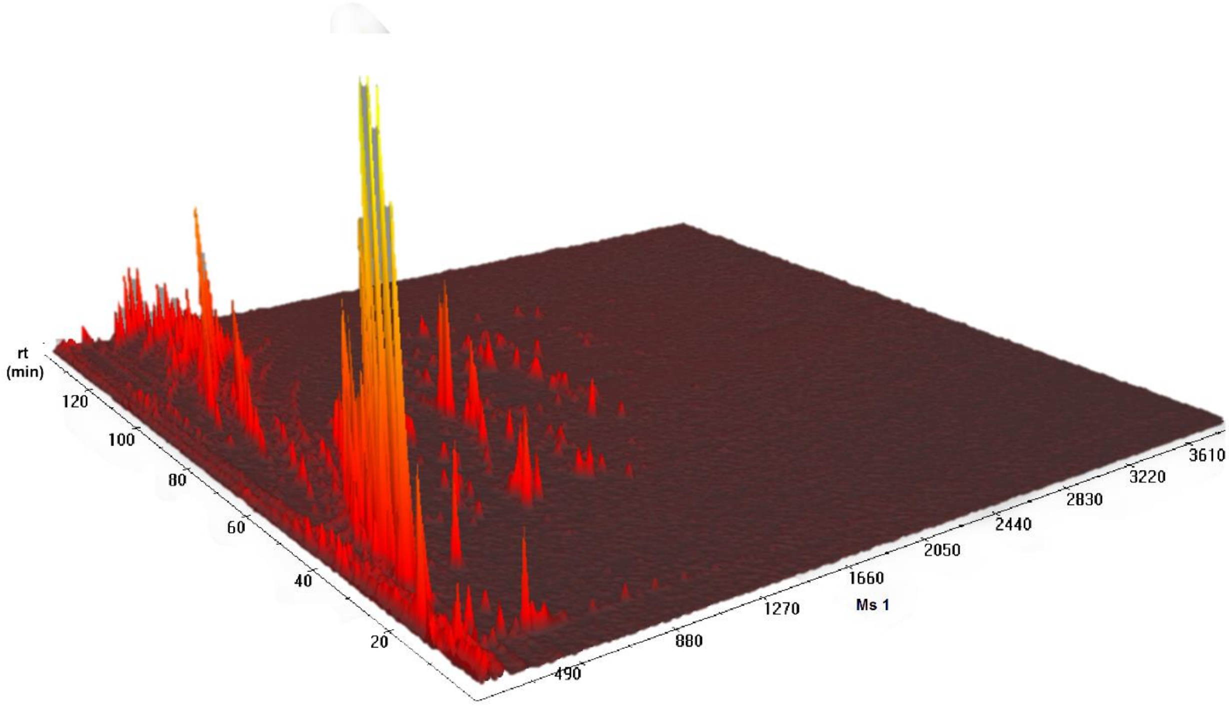
rt (min)
120
100
80
60
40
20
490
880
1270
1660
2050
2440
2830
3220
3610
Ms 1

**Supplementary Fig S2.** MS/MS sequencing of collected from the RP-HPLC of supernatant of antivenom-venom interaction. A) MS/MS fragmentation and sequencing of peptide with m/z of 1376.628 belonging to the neurotoxin with the mass of 6941 Da which obtained after trypsin digestion. B) MS/MS fragmentation and sequencing of peptide with m/z of 795.371 belonging to the Potassium channel toxin peptide with the mass of 6396 Da.

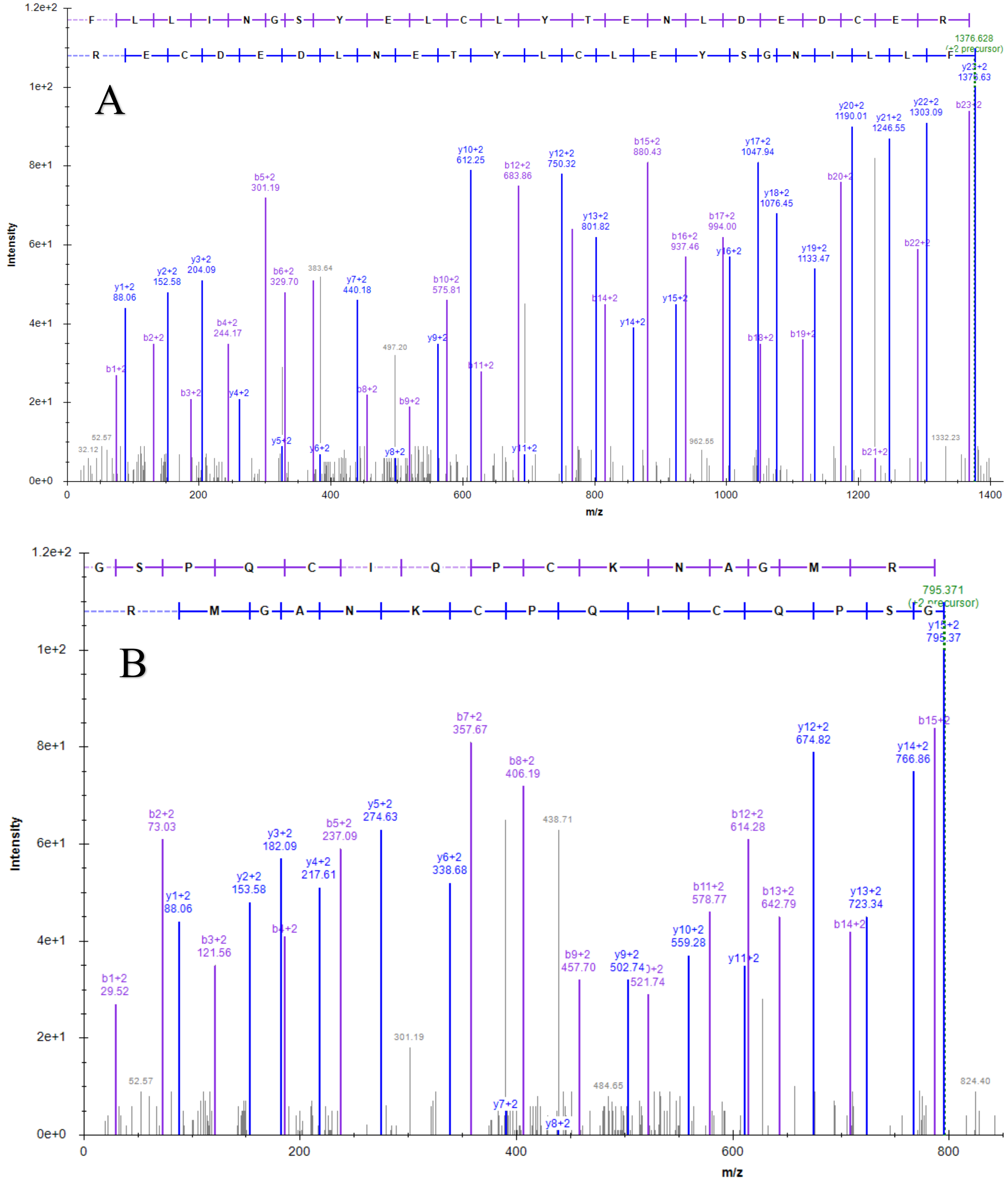

**Supplementary Table S1.** The list of identified proteins for the mass range below 10 kDa of *Odontobuthus doriae* venom was prepared by LC-MS/MS analysis followed by collection and library search of scorpions family protein databases (tax: 6855) NCBI and Unprot .

| Potassium channel inhibitor | | | | | |
|---|---|---|---|---|---|
| **Accession No.** | **Source organism** | **Identified peptide sequences** | **Sequence coverage (%)** | **Theoretical mass (Da)** | **Protein Description** |
| A0A088D9Q2_MESEU | Mesobuthus eupeus (Lesser Asian scorpion) (Buthus eupeus) | EHDR | 53 | 7227.50 | Potassium channel blocker pMeKTx18-3 |
| | | DCQR | | | |
| | | LFIVK | | | |
| | | KTCELVDMR | | | |
| | | FDGGVDK | | | |
| | | GNIMAFCDK | | | |
| | | | | | |
| A0A0U4FP89_ODODO | Odontobuthus doriae (Yellow Iranian scorpion) | NGK | 59 | 6395.90 | Potassium channel toxin KTx5 |
| | | PQCIQPCK | | | |
| | | VCSMIIGIHAGVPIDVK | | | |
| | | | | | |
| AFL70874.1 | Mesobuthus martensii (Manchurian scorpion) (Buthus martensii) | FAILR | 58 | 5294.28 | Potassium channel toxin |
| | | LILAVCSMK | | | |
| | | AIWTVNGTK | | | |
| | | PFAIRCATDADCSRKCPR | | | |
| | | | | | |
| G9BNN8_MESMA | Mesobuthus martensii (Manchurian scorpion) (Buthus martensii) | DADK | 52 | 5307.28 | Potassium blocker |
| | | RGFCACK | | | |
| | | WTVNGTPFAIR | | | |
| | | | | | |
| A0A143MGJ5_MESEU | Mesobuthus eupeus (Lesser Asian scorpion) (Buthus eupeus) | DDGVCNCNAk | 51 | 5944.05 | Potassium channel toxin meuK2-2 |
| | | VVEAVGCEECPK | | | |
| | | | | | |
| | | NDK | 61 | 6344.51 | |

| | | | | | |
|---|---|---|---|---|---|
| Q549D8_MESMA | Mesobuthus martensii (Manchurian scorpion) (Buthus martensii) | TIIPDMK<br>HCATQNAR<br>LYAIILIALVFNVR | | | Putative apamin-sensitive potassium channel inhibitor |
| | | | | | |
| A0A0U3QS34_ODODO | Odontobuthus doriae (Yellow Iranian scorpion) | LFTLK<br>VLIVLK<br>EAVGCEEK<br>CPMHR | 61 | 5972.16 | Potassium channel toxin KTx6 |
| | | | | | |
| B8XH33 | Buthus occitanus israelis | MAK<br>KPTCYDR<br>GVCNR<br>CNVK | 60 | 6306.67 | Full=Potassium channel toxin alpha-KTx 9.9 |
| | | | | | |
| A0A088DAZ8_MESEU | Mesobuthus eupeus (Lesser Asian scorpion) (Buthus eupeus) | LFTLK<br>RVLIVLAMNK<br>VMMAIIR<br>MYCK | 62 | 5926.13 | Potassium channel blocker pMeKTx2-1 |
| | | | | | |
| A0A088DB40_MESEU | Mesobuthus eupeus (Lesser Asian scorpion) (Buthus eupeus) | AYLVAK<br>VLVLR<br>SNEAVPR | 54 | 6871.22 | Potassium channel blocker pMeKTx22-1 |
| | | | | | |
| A0A218QXT6.1 | Tityus serrulatus (Buthus eupeus) | AYLVAR<br>VLVLK<br>KSNEAVPKK<br>TGGCPFSDR | 54 | 6710.08 | Full=Putative potassium channel toxin Ts21 |
| | | | | | |
| A0A0K0LBY5_9SCOR | Androctonus bicolor | DADR<br>IWTVNGTAIAIR<br>RGFCACKK | 53 | 5929.28 | Potassium channel blocker AbTx6 |
| | | | | | |
| A0A0K0LC01_9SCOR | Androctonus bicolor | IWTVNGTAIAIR<br>RGFCACK | 54 | 5945.19 | Potassium channel blocker AbTx8 |
| | | | | | |
| A0A0K0LBZ3_9SCOR | Androctonus bicolor | IFFKK<br>IWTVNGTAIAIR<br>RGFCACK | 54 | 5944.25 | Potassium channel blocker AbTx4 |
| | | | | | |
| A0A143MHF1_MESEU | Mesobuthus eupeus (Lesser Asian scorpion) (Buthus eupeus) | FAILLILAVCSMAIK<br>KLLVCPNDLFCAR | 57 | 6031.43 | Potassium channel toxin meuK5 |

| | | | | | |
|---|---|---|---|---|---|
| | | | | | |
| A0A146CJ83_MESEU | Mesobuthus eupeus (Lesser Asian scorpion) (Buthus eupeus) | VVIPK<br>VPFSTLIWK<br>TADGPSFPCR | 64 | 3883.63 | Potassium channel inhibitor |
| | | | | | |
| F1CJ17_HOTJU | Hottentotta judaicus (Black scorpion) (Buthotus judaicus) | CHK<br>KCLCMK<br>GFCLGPNGNEK | 84 | 2757.23 | Potassium channel inhibitor |
| | | | | | |
| | | | | | |
| A0A088D9S4_MESEU | Mesobuthus eupeus (Lesser Asian scorpion) (Buthus eupeus) | ECHK<br>DYYK<br>VLVLIALSAIR<br>KNATFVSCDGGYR | 51 | 6921.15 | Potassium channel blocker pMeKTx6-3 |
| | | | | | |
| A0A0K0LC08_9SCOR | Androctonus bicolor | EAK<br>WCEDHCK<br>SEYACPVIEK | 52 | 5163.08 | Potassium channel blocker AbKTx-4 |
| | | | | | |
| A0A088DAC7_MESEU | Mesobuthus eupeus (Lesser Asian scorpion) (Buthus eupeus) | DCQK<br>LFIVLVLFCILQFDR<br>TEHGK<br>CVIEK | 62 | 7126.40 | Potassium channel blocker pMeKTx18-1a |
| | | TCELVNMNGIKK | | | |
| | | | | | |
| A0A088D9R6_MESEU | Mesobuthus eupeus (Lesser Asian scorpion) (Buthus eupeus) | TCELVNMNGIKK<br>LFIVLVLFCILQFDR | 62 | 7473.83 | Potassium channel blocker pMeKTx27-1 |
| | | | | | |
| A0A0K0LBY7_9SCOR | Mesobuthus eupeus (Lesser Asian scorpion) (Buthus eupeus) | AKK<br>VEAVSCEDCPK<br>IALVLNVIMTITPDSKR | 52 | 6255.35 | Potassium channel blocker AbTx21 |
| | | | | | |
| V9LLQ5_MESMA | Mesobuthus martensii (Manchurian scorpion) (Buthus martensii) | ICQGKK<br>IFFAVLVILVLFSMLIWTAYGTPYK<br>RDCVMCGK | 63.98 | 6145.43 | Toxin BmP08 |
| | | | | | |
| Sodium channel inhibitor | | | | | |
| **Accession No.** | **Source organism** | **Identified peptide sequences** | **Sequence coverage** | **Theoretical mass (Da)** | **Protein Description** |

| | | | | | |
|---|---|---|---|---|---|
| | | | (%) | | |
| F8THJ6_HOTJU | Hottentotta judaicus (Black scorpion) (Buthotus judaicus) | GGKK<br>TEVPNR<br>DPK | 85.1 | 2244.51 | Beta-buthitoxin |
| | | | | | |
| Q6V4Y7_CENNO | Centruroides noxius (Mexican scorpion) | GYCK<br>TPTK<br>CWCK<br>CFWLGK<br>EGYLVNSTR<br>NEGCDK | 51.56 | 7049.96 | Beta-toxin |
| | | | | | |
| | | | | | |
| | | | | | |
| A0A0U4PXV5_ODODO | Odontobuthus doriae (Yellow Iranian scorpion) | CKSGR<br>DAYIAQNYK | 43.47 | 5211.02 | Sodium channel toxin NaTx5 |
| | | | | | |
| V9P3R2_MESEU | Mesobuthus eupeus (Lesser Asian scorpion) (Buthus eupeus) | LCKK<br>TDYR<br>KRPTTR<br>DMPDK<br>SDGFCRK<br>QPHCFCK<br>FLLINGSYELCLYEENLDEDCK | 68.29 | 9674.11 | Neurotoxin |
| | | | | | |
| V9P3B8_MESEU | Mesobuthus eupeus (Lesser Asian scorpion) (Buthus eupeus | ERK<br>LCKK<br>DMPDK -<br>KRPTTR<br>SDGFCRK<br>QPHCFCK<br>FLLINGSYELCLYEENLDEDCR | 64.63 | 9644.09 | Neurotoxin |
| | | | | | |
| B8XGZ1_BUTOS | Buthus occitanus israelis (Common yellow scorpion) (Buthus israelis) | NGFIAEPHK -<br>MNHLVMK<br>ALLLMTGGESVRK | 63.29 | 8609.25 | Putative alpha toxin Tx629 |
| | | CWCNGLPNNVPIVDKK | | | |
| | | | | | |
| F1CJ62_HOTJU | Hottentotta judaicus (Black scorpion) (Buthotus judaicus) | ILSVR<br>LLLALFIK<br>CSLVGWSEAVITNK | 82.75 | 3153.84 | U8-buthitoxin-Hj2a |
| | | | | | |
| A0A0K0LCH8_9SCOR | Androctonus bicolor | HDGCK<br>IWCVFRR<br>LAFSSLILTGVLTKR | 61.36 | 4935.82 | Sodium channel blocker AbNaTx23 |
| | | | | | |
| B8XH53_BUTOS | Buthus occitanus israelis (Common | MDNYK<br>DDALEELDNK | 68.08 | 5600.91 | Putative toxin Tx65 |

| | | | | | |
|---|---|---|---|---|---|
| | yellow scorpion) (Buthus israelis) | LDLDDYFDLEK<br>KPADFVLLDMR<br>MDNYR | | | |
| | | | | | |
| F1CJ97_HOTJU | Hottentotta judaicus (Black scorpion) (Buthotus judaicus) | LALFICSLVGWSEAEITNESCR | 72.41 | 3183.83 | U8-buthitoxin-Hj4a |
| | | | | | |
| A0A5P8U2Q6_MESEU | Mesobuthus eupeus (Lesser Asian scorpion) (Buthus eupeus) | LCKK<br>EQNASDGFCK<br>DMPDK<br>CERR<br>QPHCFCK | 59.93 | 6940.73 | Neurotoxin (sodium channel inhibitor) |
| | | | | | |
| | | | | | |
| F1CIZ0_HOTJU | Hottentotta judaicus (Black scorpion) (Buthotus judaicus) | PVRR<br>FSYK<br>LFSMAIWSADGTK | 73.33 | 4820.34 | U7-buthitoxin-Hj2a |
| | | | | | |
| F1CIV5_HOTJU | Hottentotta judaicus (Black scorpion) (Buthotus judaicus) | NDKK<br>CVCER<br>CEDCPER<br>LYAIILIVLVMNVIMK | 54.23 | 6630.92 | Gamma-buthitoxin-Hj1a |
| | | | | | |
| F1CJ49_HOTJU | Hottentotta judaicus (Black scorpion) (Buthotus judaicus) | EVVK<br>SGRR<br>YGNACR<br>CINLK<br>SGYCK<br>INYLMTITCAFILMTGK<br>RDAYVGDLXNCPYVCLSNSYCDGLCIEHGAK<br>EVVR<br>SGRR | 73.56 | 9721.48 | U5-buthitoxin-Hj1a |
| | | | | | |
| F1CIV0_HOTJU | Hottentotta judaicus (Black scorpion) (Buthotus judaicus) | FFQK<br>FGQPMEHQFEHSVVIVTIAPIGIVLPSVIPK | 68.42 | 6558.73 | U7-buthitoxin-Hj1a |
| | | | | | |
| Q6IZE0_MESMA | Mesobuthus martensii (Manchurian scorpion) (Buthus martensii) | GCWCK<br>CQWLGKK<br>CNDLCK<br>IELPDNVPIRK | 57.59 | 7426.52 | Neurotoxin Mm2 |
| | | | | | |
| ADY39628.1 | Hottentotta judaicus (Black scorpion) (Buthotus judaicus) | ILSVLLLK<br>CHCYPDK<br>FEYLQVKK<br>WSEA EIR | 61.29 | 3183.83 | U9-buthitoxin-Hj4a |
| | | | | | |
| A0A0U4QVR7_ODODO | Odontobuthus doriae (Yellow Iranian scorpion) | LQCK<br>DEEEK<br>FCQK | 56.96 | 9148.61 | Toxin Tx1 |

| | | | | | |
|---|---|---|---|---|---|
| | | NCTATAIKR<br>FFFNFLFSVK<br>FSYSMSSEVTAKK<br>NCTATAIKK | | | |
| | | | | | |
| F1CIX7_HOTJU | Hottentotta judaicus (Black scorpion) (Buthotus judaicus) | QCLK<br>MCMPCFTR<br>CNRR<br>CGGNGKK | 61.29 | 3340.96 | U9-buthitoxin-Hj2a |
| | | | | | |
| | | | | | |
| | | | | | |
| | | | | | |
| | | | | | |
| Chloride channel inhibitor | | | | | |
| **Accession No.** | **Source organism** | **Identified peptide sequences** | **Sequence coverage (%)** | **Theoretical mass (Da)** | **Protein Description** |
| R4H559_MESEU | Mesobuthus eupeus (Lesser Asian scorpion) (Buthus eupeus) | QCLNRK<br>AMMCMPCFTK<br>CGGNGKCFK | 56.41 | 4300.99 | Chloride channel toxin-like peptide 1b |
| | | | | | |
| Antimicrobial peptide | | | | | |
| **Accession No.** | **Source organism** | **Identified peptide sequences** | **Sequence coverage (%)** | **Theoretical mass (Da)** | **Protein Description** |
| Q685G7_MESGB | Mesobuthus gibbosus (Mediterranean checkered scorpion) (Buthus gibbosus) | MGRK<br>GFPPK<br>SHCRK | 65.38 | 2782.18 | 4kD defensin |
| | | | | | |
| A0A0K0LBU6_9SCOR | Androctonus bicolor | FRGCK<br>GGACK | 70.58 | 1938.27 | Defensin-2 |
| | | | | | |
| Q685G4_MESGB | Mesobuthus gibbosus (Mediterranean checkered scorpion) (Buthus gibbosus) | DTMVEAGFK | 61.53 | 2791.26 | 4kD defensin |
| | | | | | |
| Q685G5_MESGB | Mesobuthus gibbosus (Mediterranean checkered scorpion) (Buthus gibbosus) | APR-DTMVEAGF | 42.07 | 2859.23 | 4kD defensin(Predicted) |
| Q684T7_MESGB | Mesobuthus gibbosus (Mediterranean checkered scorpion) (Buthus gibbosus) | GACK<br>HCRK<br>MGRK | 42.03 | 2668.03 | 4kD defensin (Predicted) |
| | | | | | |
| A0A0U4L8J8_ODODO | Odontobuthus doriae (Yellow Iranian scorpion) | DFVLLK<br>DMWANMLK<br>RESSDFDDDFE<br>TLLVLK<br>LLVGK | 76.36 | 8527.42 | AMP5 |

| | | | | | |
|---|---|---|---|---|---|
| | | VLVK<br>RSTFFT<br>ADAYR | | | |
| | | | | | |
| Q684V8_MESEU | Mesobuthus eupeus (Lesser Asian scorpion) (Buthus eupeus) | LLQK<br>MEAPR<br>RGFGC | 73.07 | 2729.19 | 4kD defensin (Predicted) |
| | | | | | |
| Q684S6_MESGB | Mesobuthus gibbosus (Mediterranean checkered scorpion) (Buthus gibbosus) | VTEAK<br>RGFGC<br>QFVCDNHCKK | 74.05 | 2847.35 | 4kD defensin (Predicted) |
| | | | | | |
| Q685G6_MESGB | Mesobuthus gibbosus (Mediterranean checkered scorpion) (Buthus gibbosus) | VTEAK<br>GFGCPLFQCGCDKK | 73.08 | 2775.31 | 4kD defensin (Predicted) |
| | | | | | |
| Q684U2_MESGB | Mesobuthus gibbosus (Mediterranean checkered scorpion) (Buthus gibbosus) | TMVEAK<br>GFGGPPFK<br>QGVCDSHCRK | 80.76 | 2696.09 | 4kD defensin(Predicted) |
| | | | | | |
| Q685I0_MESGB | Mesobuthus gibbosus (Mediterranean checkered scorpion) (Buthus gibbosus) | VTEAK<br>GFGK<br>RQGSC<br>KSHCR | 65.38 | 2746.16 | 4kD defensin(Predicted) |
| | | | | | |
| A0A0K0LBU7_9SCOR | Androctonus bicolor | CDER<br>AFIVIAMLIVFVMLEEVK<br>GFVCGHCHVK<br>VQLPLSCHCPPDSKK | 61.04 | 8523.83 | Defensin-3 |
| | | | | | |
| A0A8I3B021_TITST | Tityus stigmurus (Brazilian scorpion) | FFSLIPSLVGGLISR | | 1907.51 | Stigmurin |
| | | | | | |
| E4VNZ6_MESEU | Mesobuthus eupeus (Lesser Asian scorpion) (Buthus eupeus) | TLLVLK<br>LLVGVK<br>LVSTFFTADR<br>AYPAMDNYK<br>EDLDNLK | 69.86 | 8263.18 | Venom anionic peptide-2 |
| | | | | | |
| | | | | | |
| Metallothionein | | | | | |
| **Accession No.** | **Source organism** | **Identified peptide sequences** | **Sequence coverage (%)** | **Theoretical mass (Da)** | **Protein Description** |
| C9X4I1_TITDI | Tityus discrepans (Venezuelan scorpion) | GNNR<br>MPCSCGSK<br>GGSCK<br>KCPNP | 66.66 | 4417.15 | Metallothionein |

| | | | | | |
|---|---|---|---|---|---|
| | | | | | |
| A0A0U4I1L6_ODODO | Odontobuthus doriae (Yellow Iranian scorpion) | NGDK<br>MPCSCGSR<br>GQCSCCGAKK | 68.44 | 4428.09 | Metallothionein |
| | | | | | |
| Putative zinc-finger containing protein | | | | | |
| **Accession No.** | **Source organism** | **Identified peptide sequences** | **Sequence coverage (%)** | **Theoretical mass (Da)** | **Protein Description** |
| F1CJ93_HOTJU | Hottentotta judaicus (Black scorpion) (Buthotus judaicus) | WLQLK<br>NSLTNVKK | 68.98 | 3500.09 | Transcription factor |
| | | | | | |
| | | | | | |
| Putative lipolysis-activating protein beta chain | | | | | |
| **Accession No.** | **Source organism** | **Identified peptide sequences** | **Sequence coverage (%)** | **Theoretical mass (Da)** | **Protein Description** |
| F1CJ52_HOTJU | Hottentotta judaicus (Black scorpion) (Buthotus judaicus) | YSGK<br>MHAIK<br>GFRK<br>YPDYPRK<br>IATR<br>KSLVFG | 62.5 | 5588.44 | lipolysis-activating peptides (LVPs) |
| | | | | | |
| Hemocyanin | | | | | |
| **Accession No.** | **Source organism** | **Identified peptide sequences** | **Sequence coverage (%)** | **Theoretical mass (Da)** | **Protein Description** |
| Q7M488_ANDAU | Androctonus australis (Sahara scorpion) | TVKK<br>QDRK<br>RPLFE | 71.42 | 1716.01 | Hemocyanin chain 2 |
| | | | | | |
| Q7M486_ANDAU | Androctonus australis (Sahara scorpion) | LPLFK | 42.85 | 1657.89 | Hemocyanin chain 5A |
| | | | | | |
| Putative toxin | | | | | |
| **Accession No.** | **Source organism** | **Identified peptide sequences** | **Sequence coverage (%)** | **Theoretical mass (Da)** | **Protein Description** |
| B8XH49_BUTOS | Buthus occitanus israelis (Common yellow scorpion) (Buthus israelis) | DSCK<br>VAIVLTVLLSR<br>NDNK<br>AAGRK<br>GEICCTLMTK | 61.53 | 6822.99 | Putative toxin Tx277 |
| | | | | | |
| Cellular protein | | | | | |
| **Accession No.** | **Source organism** | **Identified peptide sequences** | **Sequence coverage (%)** | **Theoretical mass (Da)** | **Protein Description** |
| A0A0K0LC85_9SCOR | Androctonus bicolor | YMKK<br>AKLDFK | 70.01 | 1200.42 | Cellular protein AbCp-62 |
| | | | | | |

| | | | | | |
|---|---|---|---|---|---|
| A0A0K0LCL3_9SCOR | Androctonus bicolor | YGYK<br>VVKK | 68.42 | 2268.57 | Cellular protein AbCp-62 |
| | | | | | |
| A0A0K0LC97_9SCOR | Androctonus bicolor | LFFLCISSMGVYPILIAGK | 90.32 | 2100.61 | Cellular protein AbCp-68 |
| | | | | | |
| | | | | | |
| | | | | | |
| | | | | | |
| | | | | | |
| | | | | | |
| | | | | | |
| Immune modulator | | | | | |
| **Accession No.** | **Source organism** | **Identified peptide sequences** | **Sequence coverage (%)** | **Theoretical mass (Da)** | **Protein Description** |
| A0A0K0LCG9_9SCOR | Androctonus bicolor | FFTADAYPSSMDK<br>NYDDALEELDNK | 70.69 | 6740.57 | Anionic peptide Aba-1 |
| | | | | | |
| E4VP16_MESEU | Mesobuthus eupeus (Lesser Asian scorpion) (Buthus eupeus) | GVLVSTFFTAK<br>KDAYPAMD<br>NYDDALEDLDNLK<br>DLDDYFR | 83.56 | 8276.22 | Venom anionic peptide-4 |
| | | | | | |
| E4VP00_MESEU | Mesobuthus eupeus (Lesser Asian scorpion) (Buthus eupeus) | SQAMEK<br>ENEEEADDSDDLR<br>DDESLDFK<br>LEDLDLK | 69.32 | 7730.30 | Venom anionic peptide-3 |
| | | | | | |
| A0A146CJB2_MESEU | Mesobuthus eupeus (Lesser Asian scorpion) (Buthus eupeus) | TLLVLLK<br>LVGVLVSTFFR<br>KTADAYPAS<br>MDNYDK<br>EPADFK<br>VLLDK | 89.18 | 8364.28 | Venom toxin meuTx19 |
| | | | | | |
| E4VNZ5_MESEU | Mesobuthus eupeus (Lesser Asian scorpion) (Buthus eupeus) | VGVLK<br>RDDYFDL<br>YDDALEK<br>DLDNLDLK<br>KVSTFFTN | 61.64 | 8277.20 | Venom anionic peptide-1 |
| | | | | | |
| E4VP26_MESEU | Mesobuthus eupeus (Lesser Asian scorpion) (Buthus eupeus) | DNYDDK<br>LDDYFDLK<br>WANMLFK<br>REPTDFVLLDM<br>DDLER | 61.68 | 8307.23 | Venom anionic peptide-5 |
| | | | | | |
| | | | | | |
| Mitochondrion | | | | | |
| B2CKW0_BUTOI | | MPQMMK<br>LGWAK | 80.39 | 6188.36 | |

| | | | | | |
|---|---|---|---|---|---|
| | Buthus occitanus (Common European scorpion) | KWLVLLL<br>VFVYFLFFVK<br>VFYFLQEVEK | | | ATP synthase subunit 8 |
| | | | | | |
| A7RAB4_MESMA | Mesobuthus martensii (Manchurian scorpion) (Buthus martensii) | SWER<br>FLVEVEVMFK<br>SLGEYGGDSKK<br>MPQMMPLGWAWMK | 72.36 | 5918.11 | ATP synthase F0 subunit 8 |
| | | | | | |
| | | | | | |
| | | | | | |
| | | | | | |
| | | | | | |
| | | | | | |
| A0A890A414_MESMA | Mesobuthus martensii (Manchurian scorpion) (Buthus martensii) | MPQMK<br>KMPLGWAWMI<br>LSLVLVYFVFMK<br>RVIFYFLVEVG<br>VMFSK | 74.50 | 6016.31 | ATP synthase F0 subunit 8 |
| | | | | | |
| Q5G7A1_CENLI | Centruroides limpidus (Mexican scorpion) | MPQMK<br>RSPLGWAW<br>VSLFVVSVYR<br>KLFYLVVFYFFYFA | 67.70 | 6058.11 | ATP synthase F0 subunit 8 |
| | | | | | |
| A0A7M3UTV5_SCOTI | Scorpiops tibetanus (Scorpion) | MSPIGWLK<br>WIIFFTIFGYLK<br>FFLILFYYFFSFK<br>FVYFSEFKK | 74.50 | 6663.05 | ATP synthase F0 subunit 8 |
| | | | | | |
| Orphan peptide | | | | | |
| **Accession No.** | **Source organism** | **Identified peptide sequences** | **Sequence coverage (%)** | **Theoretical mass (Da)** | **Protein Description** |
| A0A0K0LC43_9SCOR | Androctonus bicolor | GGGGGGGK<br>RGFG<br>LILSVVMMATLAIKK | 67.64 | 6617.67 | Orphan peptide AbOp-2 |
| | | | | | |
| Toxins having unknown target | | | | | |
| **Accession No.** | **Source organism** | **Identified peptide sequences** | **Sequence coverage (%)** | **Theoretical mass (Da)** | **Protein Description** |
| C9X4H4_TITDI | Tityus discrepans (Venezuelan scorpion) | SQVRK<br>LEPPLVR | 51.61 | 3434.06 | Uncharacterized protein |
| | | | | | |
| Q8MTW8_MESMA | Mesobuthus martensii (Manchurian scorpion) (Buthus martensii) | DPAAEK<br>EAGAGRK | 51.23 | 8249.90 | Venom peptide 1 |
| A0A5P8U2Q5_MESEU | Mesobuthus eupeus (Lesser Asian scorpion) (Buthus eupeus) | CCGK<br>QCLR<br>MCMK<br>GNGQCFGK | 44.44 | 3931.61 | Toxin |

| | | | | | |
|---|---|---|---|---|---|
| | | | | | |
| C9X4H2_TITDI | Tityus discrepans (Venezuelan scorpion) | YIVVLIISIGYNLKK | 87.5 | 1808.23 | Uncharacterized protein |
| | | | | | |
| A0A1W7R954_9SCOR | Hadrurus spadix | CFAK<br>CICR<br>LGDPVPNARK<br>LMMLLMFIQLAASK | 50.36 | 6307.66 | Venom protein |
| | | | | | |
| | | | | | |
